# Effect of Methyl Jasmonate in Gene Expression, and in Hormonal and Phenolic Profiles of Holm Oak Embryogenic Lines Before and After Infection With *Phytophthora cinnamomi*

**DOI:** 10.3389/fpls.2022.824781

**Published:** 2022-03-09

**Authors:** Marian Morcillo, Ester Sales, Elena Corredoira, María Teresa Martínez, Juan Segura, Isabel Arrillaga

**Affiliations:** ^1^Departamento de Biología Vegetal, Facultad de Farmacia, Instituto de Biotecnología y Biomedicina (BiotecMed), Universidad de Valencia, Valencia, Spain; ^2^Departamento de Ciencias Agrarias y del Medio Natural, Instituto Universitario de Investigación en Ciencias Ambientales (IUCA), Universidad de Zaragoza, Escuela Politécnica Superior, Huesca, Spain; ^3^Unidad Técnica Biotecnología y Mejora Forestal, Misión Biológica de Galicia, CSIC, Santiago de Compostela, Spain

**Keywords:** biotic stress, dieback, elicitor, epigenetic memory, induced resistance, phenols, *Quercus ilex*, somatic embryogenesis

## Abstract

The dieback syndrome affecting *Quercus ilex* and other oak species impels the search for tolerant plant genotypes, as well as methods of plant immunization against such infections. Elicitation treatments can be an effective strategy to activate plant defense response and embryogenic lines represent a promising tool to generate new tolerant genotypes and also to study early markers involved in defense response. The aim of the presented work was to investigate changes in gene expression, and in hormonal and phenolic profiles induced in three holm oak embryogenic lines (ELs) elicited with methyl jasmonate (MeJA) before and after infection with the oomycete *Phytophthora cinnamomi*, which is the main biotic agent involved in this pathogenic process. The three ELs, derived from three genotypes, showed different basal profiles in all tested parameters, noting that the VA5 naïve genotype from a scape tree was characterized by a basal higher expression in *NADPH-dependent cinnamyl alcohol dehydrogenase (CAD)* and *chalcone synthase* (*CHS)* genes and also by higher caffeic acid content. Our work also identifies changes triggered by MeJA elicitation in holm oak embryogenic lines, such as increases in ABA and JA contents, as well as in levels of most of the determined phenolic compounds, especially in caffeic acid in Q8 and E00 ELs, but not in their biosynthesis genes. Irrespective of the EL, the response to oomycete infection in holm oak elicited plant material was characterized by a further increase in JA. Since JA and phenols have been described as a part of the *Q. ilex* defense response against *P. cinnamomi*, we propose that MeJA may act as an induced resistance (IR) stimulus and that in our embryogenic material induced both direct (detected prior to any challenge) and primed (detected after subsequent challenge) defense responses.

## Introduction

Holm oak (*Quercus ilex* L.) is the most abundant and representative species of the Mediterranean forests, from Portugal and Morocco to Turkey. Holm oak, however, is particularly abundant in Spain, where it occupies around 3 million ha, usually in mixed forests with *Pinus* spp., *Quercus coccifera* or *Q. suber* (Martínez et al., 2019). Besides its common presence in the Southwest natural forests of the Iberian Peninsula, *Quercus ilex* appears in ecosystems with anthropic origin and maintenance, the so-called *dehesas* in Spain, and *montados* in Portugal. These agrosilvo-pastoral systems support a diversity of ecologic and economical services, such as acorn, honey, and black truffle production (Montero et al., 2017), therefore contributing to sustainable rural development. These ecosystems also have a great landscape, and historical and cultural value, being part of the Special Areas of Conservation defined in EU Council Directive 92/43/EEC (Council of Europe, UNEP and ECNC, 1996).

Despite their great ecological and economical value, these Mediterranean ecosystems are seriously threatened by holm oak decline, a disease also affecting cork oak (*Quercus suber)*, and that is mainly caused by the oomycete *Phytophthora cinnamomi*. Abiotic stresses, such as drought, combined with biotic stresses and the low natural regeneration rates observed for these tree species (González-Rodríguez et al., 2011), result in a complex problem aggravated by climate change. The Mediterranean area has been reported to be the most prominent climate change hot-spot, along with Northeastern Europe, according to a Regional Climate Change Index (RCCI) that estimated a large decrease in mean precipitation and an increase in precipitation variability during the dry (warm) season (Giorgi, 2006). As these expected climatic conditions, as mentioned, would favor pathogen infections in holm oak roots, there is an urgent need in obtaining cork- and holm oak plant material more adapted to upcoming ecological restrictions, and with improved tolerance to *P. cinnamomi*.

With this aim, in Spain, during the past decades, coordinated projects have implemented prevention and restoration strategies to protect natural forests and dehesas, as well as the selection of tolerant individuals in infested areas. Recently, a national program for breeding and conservation of cork- and holm oak genetic resources against the decline syndrome has also been established. For exploiting all the genetic variability existing in natural populations, superior genotypes should be vegetatively propagated, but this task is hampered by the low rooting capability of oak cuttings, which is even reduced with the aging of the trees (Martínez et al., 2020, 2021). In this context, biotechnological tools such as somatic embryogenesis constitute an interesting alternative allowing clonal propagation of holm oak adult trees (Blasco et al., 2013; Barra-Jiménez et al., 2014; Martínez et al., 2017, 2020, 2021), although acclimatization of generated plants needs to be improved. Furthermore, combining somatic embryogenesis with chemical elicitors it is possible to induce resistance triggering natural plant defenses, as a complementary strategy to breeding programs developed to obtain resilient plant material (Morcillo et al., 2020). Induced resistance (IR) is a plant state associated with an enhanced ability to resist pests and pathogen attacks or abiotic stress after an inducing stimulus. The IR phenotype is mediated by direct and primed defense responses. Defense priming refers to earlier and faster or stronger activation of cellular defense responses when the plant is subsequently challenged with the biotic or abiotic stress, and it is associated with alterations that take place directly upon IR stimulation, such as epigenetic alterations, increased receptor presence, or accumulation of hormones, among others (De Kesel et al., 2021).

Phytohormones play vital roles in plant responses to biotic and abiotic stressors and elicitors (Wang et al., 2021). Among them, abscisic acid (ABA) has been described as a pivotal hormone in plant stress defense, and its interaction with other hormones, mainly jasmonic acid (JA), salicylic acid (SA) and ethylene (ET) have also been described (Ku et al., 2018; Yang et al., 2019; Deboever et al., 2020; Bharath et al., 2021). Plant induced systemic response (ISR) depends on responsiveness to JA and ethylene rather than to SA (Goellner and Conrath, 2008; Han and Kahmann, 2019; Yang et al., 2019). In recent years, exogenous application of methyl-jasmonate (MeJA) to plant *in vitro* cultures has emerged as a novel technique for inducing hyperaccumulation of secondary metabolites (Ho et al., 2018). In addition, it has been demonstrated that this elicitor activates antioxidant enzymes and upregulates the expression of defense-related genes (Ho et al., 2020). In a previous work (Morcillo et al., 2020), we tested several elicitation treatments and found that applying 50 μM MeJA in liquid medium for 3 days to holm oak embryogenic lines (ELs) increased H_2_O_2_ production after being challenged against active oomycete, without altering somatic embryos development. Since plant defense responses have been reported to be mediated by H_2_O_2_ production (Lin et al., 2005; Wang et al., 2015), and the role of reactive oxygen species (ROS) in plant resistance to fungal pathogens is well-established (Lehmann et al., 2015), the higher H_2_O_2_ content in these MeJA treated embryos indicates activation of defense mechanisms. Corroborating this, we observed lower mycelium growth rates toward control than toward MeJA-elicited holm oak somatic embryos in dual cultures confronted to *P. cinnamomi* (Morcillo et al., 2020), although the oomycete development was not inhibited.

Several studies reported on the importance of secondary metabolites, mainly phenol-derivates, for the susceptible and resistant interaction between woody species and *P. cinnamomi* (Cahill et al., 1992). Also, during pathogen attack the phenylpropanoid pathway genes were found to be overexpressed, resulting in increasing activities and accumulation of various phenolic compounds (Yadav et al., 2020). More recently, our group demonstrated that axillary shoots from tolerant holm oak genotypes established *in vitro* had higher total phenol content than other susceptible genotypes (Martínez et al., 2021).

The aim of this study was to determine whether priming holm oak embryogenic lines with MeJA modifies gene expression and hormone and phenolic profiles before and after infection with *P. cinnamomi*. For this purpose, three holm oak genotypes (E00, Q8, and VA5), one of them (VA5) being a tolerant tree selected in our breeding program, were tested.

## Materials and Methods

### Plant and Oomycete Material

The experiments were undertaken with three ELs established from the E00, Q8, and VA5 holm oak genotypes. Embryogenic lines E00 and Q8, generated as described in Barra-Jiménez et al. (2014), were kindly provided by Dr. M. Toribio from IMIDRA (Instituto Madrileño de Investigación y Desarrollo Rural, Agrario y Alimentario, Madrid, Spain), while the VA5 line was established in our lab from male catkins of an “escape” tree, following the protocols described in Blasco et al. (2013). The genotype VA5 is a 50–100-year-old holm oak located in Mount of Vallivana (Castellón, Spain, 40°31'60”N, 0°1'0”E) that grows in soil naturally infested with *Phytophthora cinnamomi* and does not present symptoms of decline. Genotypes Q8 (Quintos de Mora, Los Yémenes, Toledo, Spain, 39°24'23”N, 4°4'19”W) and E00 (El Encín, Alcalá de Henares, Madrid, Spain, 40°3'02”N, 3°17'11”W) have not been previously exposed to *P. cinnamomi*, since they grow in an area not affected by the oomycete. The three ELs were routinely maintained in MS (Murashige and Skoog, 1962) medium supplemented with 20 μM silver thiosulfate and 4 g/L activated charcoal (named after MS/STS/AC medium), conditions that induced secondary embryogenesis, as described by Martínez et al. (2015).

*Phytophthora cinnamomi* strain 1,630 was kindly provided by Dr. P. Abad (group Phytopathogenic fungi, Instituto Agroforestal Mediterráneo—Universidad Politécnica de Valencia, Spain) and was maintained in PDA medium (Potato Dextrose Agar, Pronadisa, Spain) by subculturing mycelium pieces of 0.5 cm^2^ to fresh medium every 15 days.

### Elicitation and Infection Treatments

For each holm oak embryogenic line, three aliquots of about 1 g of plant material containing somatic embryos at the globular stage were cultured for 3 days in 250 mL Erlenmeyer flasks containing 40 mL of Elicitin Secretion Medium (ESM, Horta et al., 2008) supplemented with 50 μM MeJA, as described in Morcillo et al. (2020). After this period, plant material was recovered by filtering and was transferred to MS/STS/AC medium.

Suspensions of *P. cinnamomi* strain 1,630 were prepared by inoculating in flasks with 40 mL of ESM, 5 sections of 0.5 cm^2^ of mycelium, taken from 10-days-old PDA cultures. Flasks were then incubated under agitation (50 rpm) and in the dark, in a growth chamber at 23 ± 2°C for 4 days. Subsequently, oomycete cultures were filtered (Whatman^®^ paper N2) and the liquid extract was diluted to 20% (v/v) with ESM. Samples (1 g) of control and elicited embryogenic material, cultivated in MS/STS/AC medium for 14 days, were immersed for 3 h in 40 mL of this oomycete suspension, and then recovered by filtering and transferred again to solid plates containing MS/STS/AC medium. After 24 h, plant material was either frozen in liquid nitrogen or lyophilized, and stored at −80°C until analysis. For each line and treatment, 3 replicates were prepared.

### Gene Expression Analysis

We investigated by qPCR the expression of four genes coding for key enzymes involved in phenolic compounds synthesis: *chorismate synthase* (*CS*), *phenylalanine-ammonia-lyase* (*PAL*), *NADPH-dependent cinnamyl alcohol dehydrogenase* (*CAD1*), and *chalcone synthase* (*CHS*), using primers designed for *Quercus suber* by Chaves et al. (2011). An elongation factor (*EF*) was used as a reference gene, with primers designed by Soler et al. (2008). Total RNA isolation from 0.1 g of frozen aliquots of holm oak embryogenic cell lines was performed using the Plant and Fungi RNA isolation kit (Norgen Biotek, Thorold, Canada). After treatment with DNase (Takara Bio Inc., Japan), 1 μg of RNA from each sample was used to synthetize cDNA with a reverse transcriptase kit (Takara Bio Inc., Japan). Amplifications were performed as described in Martínez et al. (2021). Gene expression was estimated by the 2^−ΔCT^ method described by Pfaffl (2001).

### Hormone Content Determinations

The content in three hormones regulating biotic stress response (ABA, JA, and SA) was determined using lyophilized samples (25 ± 3 mg) of control and elicited and/or infected holm oak ELs. Homogenized material was extracted with 80% methanol acidified with 1% acetic acid. At this point, internal standards (deuterium-labeled hormones from OlChemim Ltd., Olomouc, Czeck Republic), were also added. Extracts were obtained in agitation at 4°C for 1 h, then were maintained overnight at −20°C, and then evaporated under vacuum. Dry residues were dissolved in 1% acetic acid aqueous solution and injected in a reverse phase column (HLB Oasis 30 mg, Waters Corporation, USA), as described by Seo et al. (2011). After that, samples were dried again and finally resuspended in 5% acetonitrile acidified with 1% acetic acid. These extracts were analyzed by LC/MS/MS (Instituto de Biología Molecular y Celular de Plantas, IBMCP, Valencia) using a Q-Exactive Orbitrap^TM^ (Thermo Fisher Scientific, USA) that was equipped with a reverse phase column Accucore C18 (2.6 mm, 100 mm; Thermo Fisher Scientific, USA), and coupled to a mass spectrometer. Hormones were separated in a gradient of 2–55% acetonitrile acidified with 0.05% acetic acid, with a flow rate of 0.4 mL/min for 21 min. Analysis conditions were as follows: capillar temperature, 300°C; ionization spray voltage 3.0 kV; heater temperature, 150°C; gas flow 40 mL/min; auxiliary gas flow, 10 mL/min; negative mode. Contents in ABA, JA, and SA were determined using integrated calibration curves and software Xcalibur 4.0 and TraceFinder 4.1 SP1.

### Phenolic Content Determinations

Samples (0.1 mg) of lyophilized material from control, elicited and infected E00, Q8, and VA5 ELs were extracted with 1 mL of 80% methanol for 30 min in an ultrasonic bath. Three replicated samples were prepared for each line and treatment. After being filtered (0.22 μm), extracts were analyzed by UPLC/QTOF. Analyses were performed in a UPLC-QTOF system (ABSciex TripleTOF™ 5600 LC/MS/MS, Servicio Central de Apoyo a la Investigación Experimental, SCSIE, Universidad de Valencia) with a Waters UPLC C18 column (50 mm x 2.1, 1.7 μm). The mobile phase was a gradient of methanol and water (Table 1), both acidified with 0.1% formic acid, at a flow rate of 0.4 mL/min. Accumulating time was 100 ms, and ionization conditions were: ion source 50 psi, curtain gas 1:25 psi, temperature 400°C, voltage (ISVF)−4500, and collision energy−50. Acquisition data was performed in negative mode, in a mass range of 80–1,200 m/z.

**Table 1:** Gradient of elution used for separating phenolic compounds by UPLC/QTOF.

**Time (min)**	**H_2_O** **0.1% Formic acid**	**MeOH** **0.1% Formic acid**
0	90	10
2	90	10
13	0	100
15	0	100
15.1	90	10
22	90	10

Among the detected compounds, it was possible to quantify four phenolic acids (caffeic, ellagic, ferulic and sinapic), the flavanol quercetin, and a proanthocyanidin, prodelphinidin B3. We obtained calibration curves using 6 serial dilutions (25–1,000 mg/mL) of reference standards of the four acids (Sigma- Aldrich, USA), and a reference standard of quercetin-3-O-glucoside (Extrasynthese, Genay Cedex, France).

### Statistical Analysis

Data were analyzed by analysis of variance (ANOVA), and are presented as mean ± standard error of three independent replications. When appropriate, treatment means were separated using *t*-test or Tukey's HSD. Data that not followed a normal distribution were analyzed by the Kruskal-Wallis non-parametric ANOVA and values distributions pairwise comparison. Correlation coefficients were also estimated among the determined parameters. All statistical analyses were performed using SPSS for Windows, version 26 (SPSS Inc., Chicago, IL, USA). A principal component analysis was performed using R software (R Core Team, 2020).

## Results

### Gene Expression Patterns in Elicited and Infected Holm Oak Embryogenic Material

Our qPCR analysis of the transcription of four genes regulating phenols biosynthesis in holm oak ELs showed significant differences in the expression patterns that varied among lines and treatments, but results depended on the studied gene. When averaged among treatments, expression of the *CS* gene varied among genotypes (*p* < 0.001), and the response to infection and elicitation treatments also differed in each holm oak line. Basal expression of the *CS* gene depended on the line (*p* = 0.002), since control samples of ELs from genotype E00 showed significantly lower expression levels than those from Q8 and VA5 (Table 2). Furthermore, these low expression levels in E00 control plant material were not affected by elicitation with MeJA and/or infection with *P. cinnamomi*, while these treatments affected *CS* gene expression in the other two lines (*p* = 0.046 and *p* = 0.011 for Q8 and VA5, respectively). However, significant differences were observed only after infection of control or elicited Q8 embryogenic material, and after elicitation in the VA5 line, in which this treatment reduced *CS* gene expression but levels were recovered after infection (Table 2). Expression pattern of the *PAL* gene did not vary significantly among lines but was significantly affected by treatment (*p* = 0.014), since infection with *P. cinnamomi* induced higher levels of expression of this gene, particularly in elicited material (2-fold increase). In contrast, *CAD* gene expression (Table 2) significantly differed among lines (*p* = 0.016), since on average it was significantly lower in E00 (2^−ΔCT^ = 0.27 ± 0.04) than in VA5 (2^−ΔCT^ = 0.44 ± 0.02), the values of which did not differ from those determined in plant material from the Q8 line (2^−ΔCT^ = 0.36 ± 0.02). Elicitation and infection treatments did not affect *CAD* expression in the three analyzed holm oak ELs. Finally, *CHS* gene expression pattern varied among lines (*p* = 0.049), being on average significantly higher in E00 embryogenic material (2^−ΔCT^ = 0.014 ± 0.002) than in that from the Q8 line (2^−ΔCT^ = 0.009 ± 0.002), while VA5 material showed intermediate values (2^−ΔCT^ = 0.012 ± 0.005). Elicitation and infection treatments also differentially affected *CHS* gene expression in the three lines (*p* = 0.007), since it remained unchanged in E00 (Table 2), but was significantly repressed after infection in elicited material of Q8 and in control and elicited material of VA5. Specific comparations on gene expression between treatments are depiced in Supplementary Figure 1 corroborating that gene expression patterns depended on the genotype.

**Table 2:** Expression of *chorismate synthase* (*CS*), *phenylalanine-ammonia-lyase* (*PAL*), *NADPH-dependent cinnamyl alcohol dehydrogenase* (*CAD*), and *chalcone synthase* (*CHS*) genes in embryogenic lines from E00, Q8, and VA5 *holm oak* genotypes.

	**Treatment**	**E00**	**Q8**	**VA5**	**Mean**
*CS*	Control	0.005 ± 0.002	0.036 ± 0.004	0.038 ± 0.005	0.026 ± 0.011
	Infected	0.009 ± 0.002	0.056 ± 0.008	0.032 ± 0.005	0.032 ± 0.014
	Elicited	0.004 ± 0.000	0.038 ± 0.004	0.011 ± 0.002^x^	0.017 ± 0.010
	Elicited + Infected	0.009 ± 0.001	0.032 ± 0.003	0.030 ± 0.005	0.024 ± 0.008
*PAL*	Control	0.056 ± 0.018	0.103 ± 0.018	0.120 ± 0.037	0.093 ± 0.019
	Infected	0.104 ± 0.024	0.155 ± 0.025	0.116 ± 0.035	0.125 ± 0.016
	Elicited	0.044 ± 0.008	0.110 ± 0.012	0.021 ± 0.002	0.058 ± 0.027
	Elicited + Infected	0.131 ± 0.023	0.086 ± 0.015	0.135 ± 0.046	0.117 ± 0.016^y^
*CAD*	Control	0.205 ± 0.072	0.306 ± 0.090	0.450 ± 0.027	0.320 ± 0.071
	Infected	0.284 ± 0.074	0.382 ± 0.051	0.424 ± 0.019	0.363 ± 0.042
	Elicited	0.212 ± 0.026	0.398 ± 0.039	0.467 ± 0.075	0.359 ± 0.076
	Elicited + Infected	0.360 ± 0.112	0.338 ± 0.005	0.400 ± 0.154	0.366 ± 0.018
*CHS*	Control	0.017 ± 0.004	0.007 ± 0.002	0.024 ± 0.008	0.016 ± 0.005
	Infected	0.013 ± 0.002	0.007 ± 0.002	0.005 ± 0.001^y^	0.008 ± 0.002
	Elicited	0.014 ± 0.002	0.015 ± 0.003	0.015 ± 0.002	0.015 ± 0.000
	Elicited + Infected	0.012 ± 0.002	0.006 ± 0.001^y^	0.003 ± 0.001^y^	0.007 ± 0.003

*Samples were taken from control and elicited with 50 μM methyl-jasmonate embryogenic lines, before and after infection with a suspension of P. cinnamomi. Data are mean ± SE of triplicated amplifications performed with three biological replicates of each treatment and line*.

x *Significantly affected by elicitation, p < 0.05 according to t-test*.

y *Significantly affected by infection, p < 0.05 according to t-test*.

Interestingly, we estimated significant correlations among expression levels of some genes, since samples with higher transcription abundance of the *CS* gene also showed higher expression levels of both *PAL* (ρ = 0.681, *p* < 0.001) and *CAD* (ρ = 0.476, *p* = 0.003) genes. Furthermore, expression rates of these two genes also showed significant positive correlation (ρ = 0.388, *p* = 0.019).

### Hormone Profiles in Elicited and Infected Holm Oak Embryogenic Cultures

**Figure 1:**
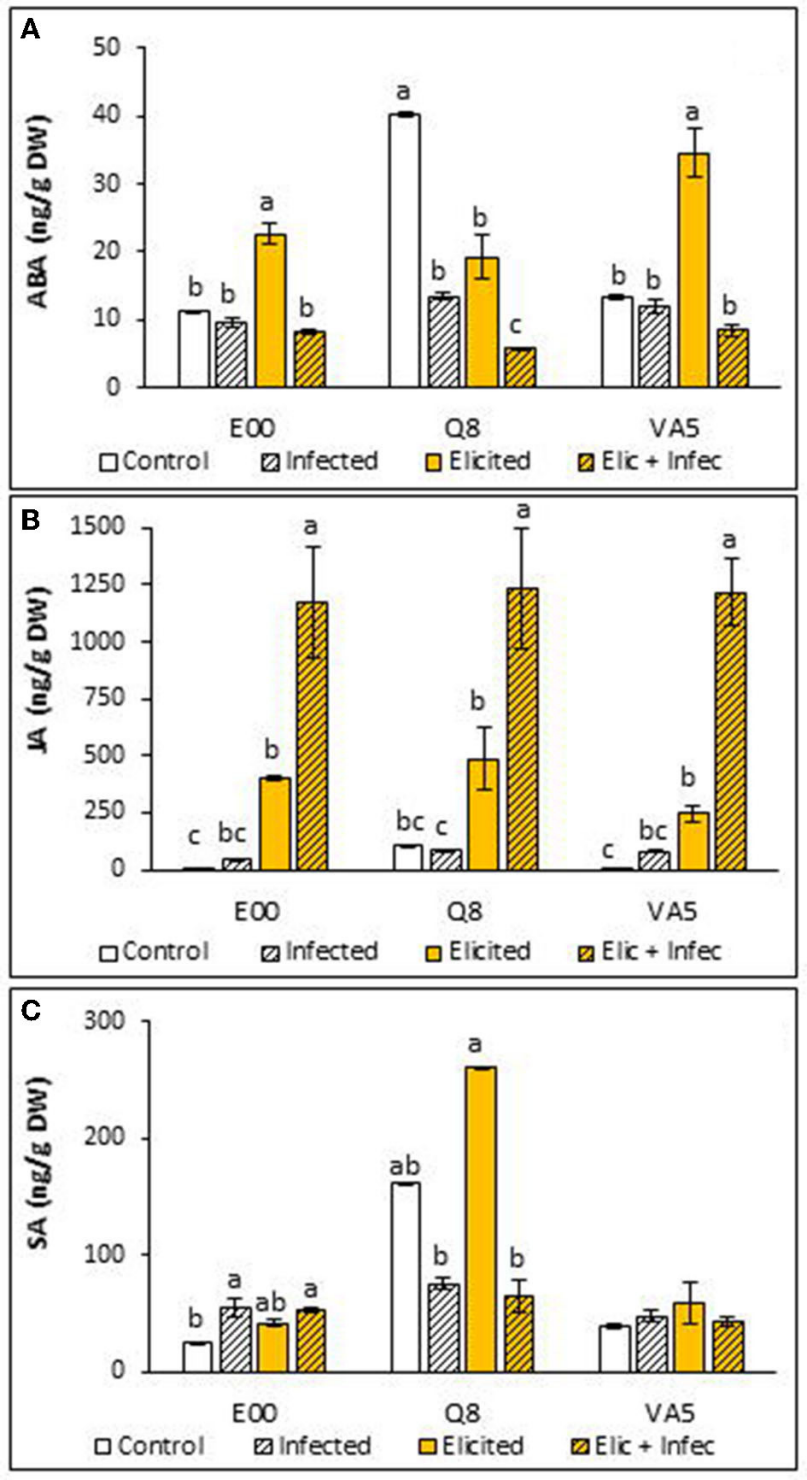
Hormones content in control and elicited with 50 μM methyl-jasmonate embryogenic lines from three *Quercus ilex* genotypes (E00, Q8, and VA5), before and after infection with *P. cinnamomi*. For each line and treatment, data are mean ± SE of abscisic acid **(A)**, jasmonic acid **(B)** and salicylic acid **(C)** determinations, performed by triplicate. Within each line, values followed by the same letter did not differ significantly (*p* < 0.05) from one another, according to distribution pairwise comparisons in a Kruskal-Wallis test.

Levels of ABA determined in *Q. ilex* embryogenic lines were on average similar among lines, but elicitation with 50 μM MeJA and infection with *P. cinnamomi* affected differentially the production of this hormone in embryogenic material from the three analyzed genotypes (Figure 1A). Non- parametric analyses showed that basal levels of ABA were significantly higher in Q8 cells than in those from E00 and VA5 genotypes (40.10 ± 0.35 ng/g DW front to 11.15 ± 0.09 and 13.25 ± 0.26 ng/g DW, respectively; *p* = 0.027). This higher content determined in Q8 ELs was significantly reduced after elicitation (2-fold decrease) and also after infection (3-fold decrease), and when both treatments were combined (9-fold decrease). The low basal levels determined in control material from E00 and VA5 were also significantly altered after elicitation and infection treatments (*p* = 0.015 and *p* = 0.024, respectively), since they were slightly reduced after infection and increased after MeJA elicitation.

We found very low basal levels of JA in holm oak ELs (on average 41.22 ± 32.59 ng/g DW), particularly in plant material from E00 and VA5 genotypes, and these levels were significantly affected by elicitation and infection treatments (*p* < 0.001). Jasmonic acid contents did not increase significantly after infection with *P. cinnamomi* (on average 70.08 ± 13.22 ng/g DW), while levels of this hormone increased significantly (about 9-fold) in embryogenic material from the three genotypes after the elicitation treatment (Figure 1B). Infection of this elicited material resulted in even higher contents of JA (3-fold increase). These changes were observed in embryogenic cells from the three analyzed genotypes, without significant differences among lines in their JA content (*p* = 0.388).

Levels of SA in holm oak ELs varied among lines (*p* < 0.001) and were also affected differentially by elicitation and infection treatments, which significantly altered SA contents of E00 (*p* = 0.043) and Q8 (*p* = 0.024) lines, but not those determined in VA5 plant material (*p* = 0.578). Basal SA levels in embryogenic material from the Q8 genotype were significantly higher than those found in material from E00 and VA5 genotypes (*p* = 0.027). Infection with *P. cinnamomi* significantly affected SA content in material from the E00 genotype, since it increased from 24.73 ± 1.64 ng/g DW determined in control samples to 54.67 ± 7.77 ng/g DW in the infected ELs, and SA levels were also significantly higher (53.27 ± 1.23 ng/g DW) in elicited and infected E00 material (Figure 1C). In contrast, after infection with *P. cinnamomi* we observed in embryogenic material from the Q8 genotype reduced SA contents, but this effect was significant only for elicited material (from 260.85 ± 0.14 to 65.40 ± 13.63 ng/g DW).

### Phenolic Content Determinations

Non-parametric one-way analysis of variance found significant differences among lines for ellagic, ferulic, and sinapic acid contents (*p* < 0.001, *p* < 0.001, and *p* = 0.009, respectively). These differences were explained by the higher levels of ellagic acid determined in E00 plant material, in contrast to those found in ELs from Q8 and VA5 genotypes, and by the lower ferulic and sinapic acids contents determined in material from the VA5 line.

**Figure 2:**
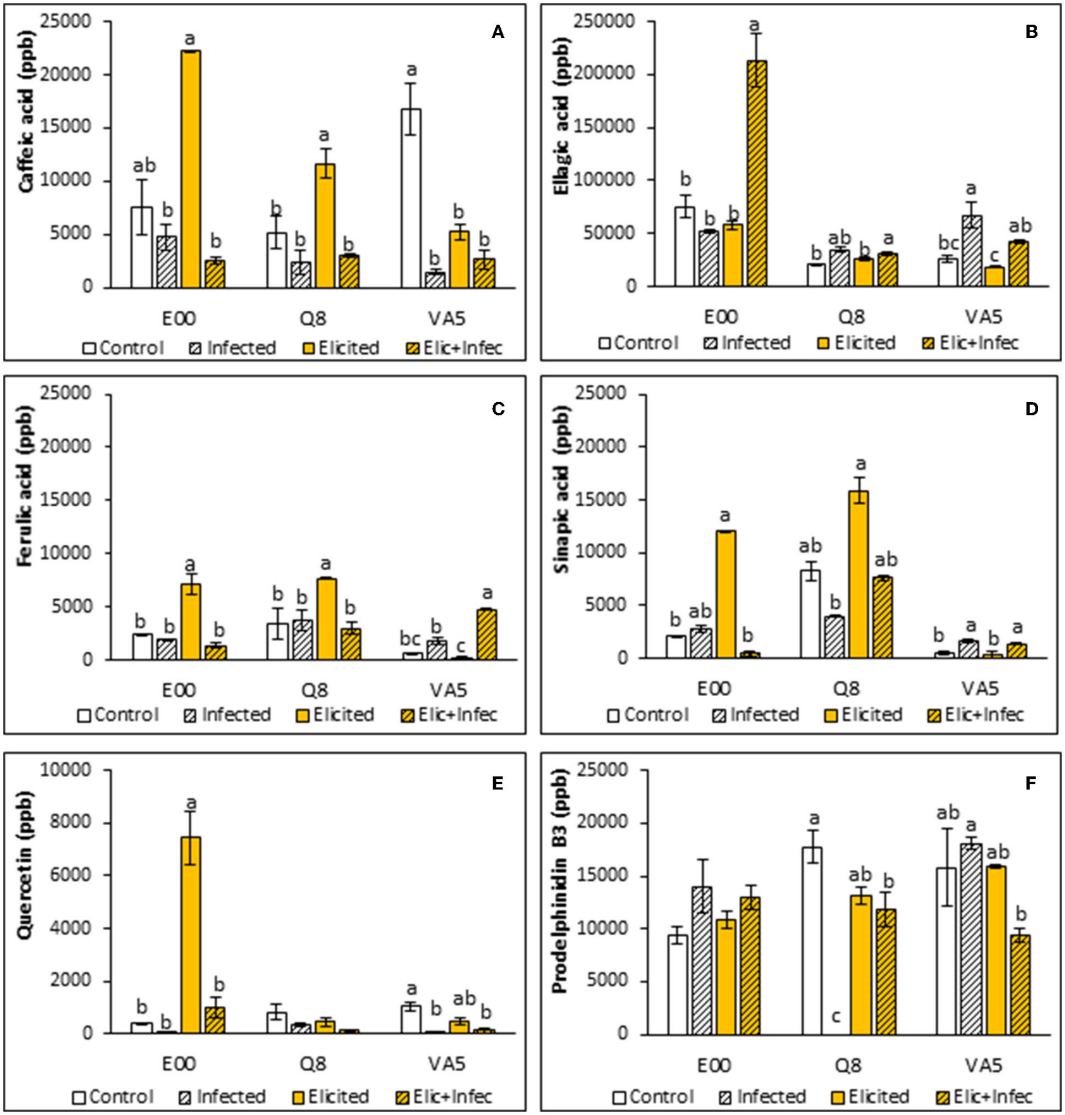
Phenolic compounds content in control and elicited with 50 μM methyl-jasmonate embryogenic material from three *Quercus ilex* genotypes (E00, Q8, and VA5), before and after infection with *P. cinnamomi*. For each line and treatment, data are mean ± SE of caffeic acid **(A)**, ellagic acid **(B)**, ferulic acid **(C)**, sinapic acid **(D)**, quercetin **(E)**, and prodelphinidin B3 **(F)** determinations, performed by triplicate. Within each line, values followed by the same letter did not differ significantly (*p* < 0.05) from one another, according to distribution pairwise comparisons in a Kruskal-Wallis test.

A Kruskal-Wallis test also revealed that caffeic acid contents of holm oak ELs were significantly affected by elicitation and infection treatments (*p* < 0.001), and the variation observed in pairwise comparisons depended on the genotype. Basal levels of caffeic acid determined in embryogenic material from the tolerant VA5 genotype were significantly higher than those found in control material from E00 and Q8 genotypes (1,6746 ± 2,364 front to 7,493 ± 2,609 and 5,194 ± 1,469 ppb, respectively, *p* = 0.022). After elicitation with 50 μM MeJA, caffeic acid content increased significantly in these two E00 and Q8 ELs (on average 2.7-fold), while decreasing in VA5 plant material in a similar rate (Figure 2A). Infection with *P. cinnamomi* reduced caffeic acid levels in both control and elicited plant material from the three genotypes (Figure 2A), but these differences were significant for control material of the VA5 genotype (11-fold decrease) and for elicited material from the E00 and Q8 lines (6-fold decrease on average).

Ellagic acid content depended on the genotype, as mentioned, and significant differences were also observed among treatments for the three ELs (*p* = 0.033, *p* = 0.034, and *p* = 0.016 for E00, Q8 and VA5 lines, respectively). Levels determined in control samples varied significantly among lines (*p* = 0.039), since those from the E00 genotype were higher than those determined in Q8 samples, while embryogenic material from the VA5 genotype showed intermediate values (Figure 2B). Irrespective of the genotype, these basal levels were not significantly affected by the elicitation treatment. The higher initial rates determined in E00 embryogenic material remained unchanged also after infection with *P. cinnamomi*. In contrast, infection increased ellagic acid content in samples (elicited or not) from Q8 and VA5 ELs, although this increase was not significant for elicited Q8 material, which contrasted with the drastic increase (3.7-fold) observed in elicited material from the E00 genotype.

Elicitation and infection treatments affected ferulic acid content of holm oak embryogenic lines, since significant differences were observed among E00 (*p* = 0.016), Q8 (*p* = 0.025) and VA5 (*p* = 0.016) samples. In E00 and Q8 ELs elicitation with 50 μM MeJA significantly increased ferulic acid contents, while the low basal levels observed in VA5 remained unaffected (Figure 2C). This differential response to the elicitation treatment was more evident in infected samples, since ferulic acid contents of control embryogenic material from the three genotypes did not change after exposure to *P. cinnamomi*, while were significantly reduced in elicited embryogenic cultures from E00 and Q8 genotypes and significantly increased in those from the VA5 genotype (Figure 2C).

Regarding sinapic acid contents, the responses of ELs to elicitation and infection treatments depended on the genotype but significant differences were also observed (*p* = 0.041, *p* < 0.001, and *p* = 0.003 for E00, Q8, and VA5 plant material, respectively). Basal sinapic acid levels were significantly different among the three analyzed lines (*p* < 0.001), since plant material from the Q8 line showed initially higher contents (8,244 ± 924 ppb) than those from E00 and VA5 genotypes (2,124 ± 53 and 504 ± 206 ppb, respectively). Elicited samples from E00 and Q8 lines showed increased levels of this acid as compared to controls (Figure 2D), but this difference was significant only for E00 plant material. The sinapic acid contents determined in embryogenic cells from the VA5 genotype were not affected by the MeJA priming treatment, while increasing significantly after infection with *P. cinnamomi*. Sinapic acid production slightly increased in infected material from the E00 genotype, while infection of elicited E00 material with *P. cinnamomi* induced a significant 25-fold decrease in this acid (Figure 2D). Finally, infection of Q8 control and elicited material slightly reduced sinapic acid levels in this line.

Quercetin contents of holm oak ELs were significantly affected by elicitation and infection treatments (*p* = 0.001), since on average levels of this flavanol decreased after infection as compared to control and elicited material, but the response depended on the genotype of the EL (Figure 2E). No significant differences were observed among samples from the Q8 line (*p* = 0.090), while elicitation and infection treatments affected quercetin contents of E00 and VA5 lines (*p* = 0.024 and *p* = 0.019). The lower levels determined in control E00 material drastically increased after elicitation with MeJA (about 18-fold), and decreased after infection with *P. cinnamomi* (about 8-fold). In contrast, infection with the oomycete significantly reduced the quercetin content of VA5 embryogenic material, that was not affected by the elicitation treatment.

Prodelphinidin B3 contents determined in holm oak ELs did not vary among lines (*p* = 0.214) and were on average not affected by elicitation and infection treatments (*p* = 0.522). However, when the ANOVA was performed separately for each genotype, we corroborated this result for the E00 genotype, but we found significant variation among treatments for the Q8 line (*p* < 0.001), since prodelphinidin B3 was not detected in infected material and decreased significantly in elicited and infected ELs from this genotype (Figure 2F). In contrast, infection with *P. cinnamomi* increased prodelphinidin B3 content in non-elicited material from the VA5 genotype, while levels of this anthocyanidin decreased after infection of MeJA treated samples.

When results from the three holm oak genotypes and four treatments were combined, we found that higher levels of ferulic acid significantly correlated with higher levels of sinapic acid (ρ = 0.761, *p* < 0.001 and with lower levels of prodelphinidin B3 (ρ = −0.385, *p* = 0.020). We also found a significant positive correlation between caffeic acid and quercetin contents (ρ = 0.499, *p* = 0.002). Phenolic compounds accumulation was also correlated to hormone contents, since higher levels of ABA were associated to higher contents in caffeic acid (ρ = 0.467, *p* = 0.004) and quercetin (ρ = 0.488, *p* = 0.003), and with lower levels of ellagic acid (ρ = −0.545, *p* = 0.001). Increases in JA contents were positively correlated with increases in ferulic acid content (ρ = 0.338, *p* = 0.044), while levels of SA showed significant positive correlation with sinapic acid content (ρ = 0.467, *p* = 0.004) and negative correlation with ellagic acid content (ρ = −0.431, *p* = 0.009). Finally, we also estimated significant correlations between phenolic compounds contents and levels of expression of genes related to phenol metabolism, since ellagic acid content was inversely correlated to higher levels of expression of *CS* (ρ = −0.476, *p* = 0.003) and *CAD* (ρ = −0.343, *p* = 0.040) genes, while expression of the *PAL* gene was inversely correlated to caffeic acid content (ρ = −0.351, *p* = 0.036). In contrast, expression of the *CHS* gene was positively correlated to caffeic acid (ρ = 0.636, *p* < 0.001) and quercetin (ρ = 0.512, *p* = 0.001) contents. Expression of this gene was inversely correlated with JA levels (ρ = −0.340, *p* = 0.042), while higher contents in SA were significantly associated with higher expression rates of the *CS* gene (ρ = 0.440, *p* = 0.007).

**Figure 3:**
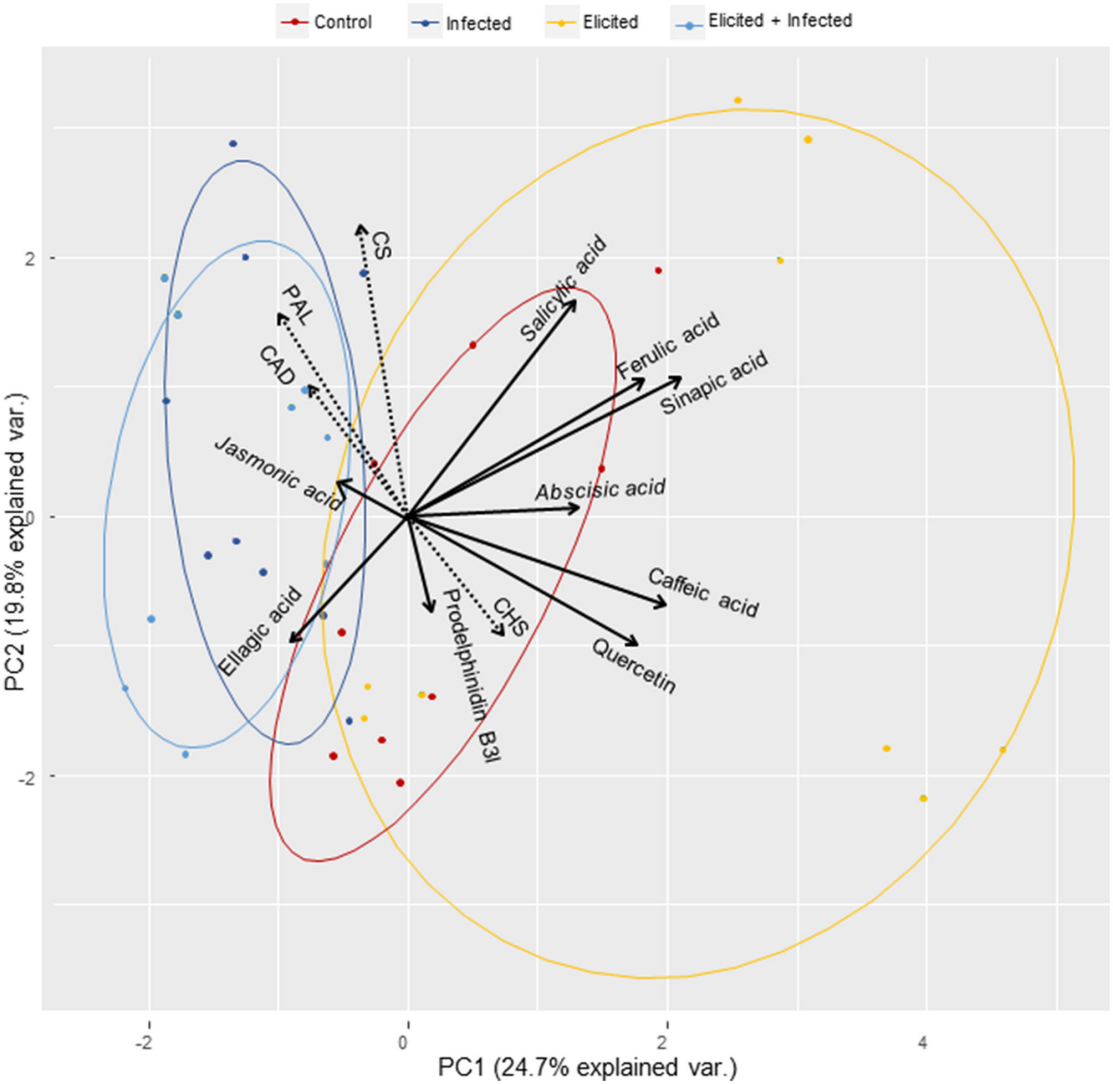
Principal component analysis of the observed variation in the expression of four genes: *chorismate synthase (CS), phenylalanine-ammonia-lyase (PAL*), *NADPH-dependent cinnamyl alcohol dehydrogenase (CAD)* and *chalcone synthase (CHS)*, and in the content of hormones (abscisic acid, jasmonic acid and salicylic acid) and phenolic compounds (caffeic acid, ellagic acid, ferulic acid, sinapic acid, quercetin and prodelphinidin B3) in *Quercus ilex* embryogenic lines after elicitation with 50 mM MeJA and/or infection with *P. cinnamomi*. For each treatment, ELs from 3 genotypes were analyzed in triplicate.

These relationships among the determined variables can be observed in a principal component analysis (PCA) that resumes variability found in holm oak ELs after the elicitation and infection treatments (Figure 3). Higher variation was found in the response of ELs to the elicitation treatment, that was characterized by increases in *CHS* gene expression and in ABA and SA contents, as well as in levels of most of the determined phenolic compounds. Infection with *P. cinnamomi* of either control or elicited holm oak ELs was mainly characterized by overexpression of *CS, PAL*, and *CAD* genes, and by higher contents in JA and, in a less extent, in ellagic acid.

## Discussion

Holm oak decline in the Southwest of the Iberian Peninsula is caused by abiotic (climate and edaphic conditions, topography, and inter and intraspecific competence) and biotic factors such as xylophagous insects and pathogens, particularly the oomycete *Phytophthora cinnamomi* (González-Rodríguez et al., 2011; Duque-Lazo et al., 2018). In this scenario, it is necessary to generate new genotypes with an increased resilience to the pathogen, as well as to develop tools to identify tolerant genotypes. With this aim, we tested whether priming holm oak embryogenic lines with MeJA induces a primed phenotype associated to changes in gene expression and in hormones and phenolic profiles, and if these profiles are modified after challenging plant material to the oomycete.

The four genes of which expression was analyzed in this work *(CS, PAL, CAD*, and *CHS*) have been reported as candidate genes in cork oak response to the oomycete (Oßwald et al., 2014), and are related to the biosynthesis of phenylpropanoids (Martínez et al., 2021). The VA5 embryogenic line, generated from a tolerant tree, displayed a basal phenotype with high expression in *PAL, CAD*, and *CHS* genes, as compared to E00 and Q8 lines. Elicitation of VA5 line with MeJA decreased or did not alter the expression of these genes while increasing *CHS* gene expression in the other genotypes (Supplementary Figure 1A). Although differences were not significant, we observed a trend of increased expression of *CS, PAL*, and *CAD* genes after infection with oomycete in lines E00 and Q8, but not in the VA5 line (Supplementary Figure 1B). Besides, the expression of the *CHS* gene was significantly repressed in infected material from the tolerant genotype, while it remained unaffected in E00 and Q8 embryogenic cells. In contrast, *PAL* and *CHS* genes were upregulated when *in-vitro* derived shoots established from the VA5 genotype were confronted for 17 days with *P. cinnamomi* in dual cultures (Martínez et al., 2021) corroborating that the transcriptional level of genes coding for *PAL* and *CHS* enzymes is tissue-dependent (Rubio-Rodríguez et al., 2021). Both, *PAL* and *CS* genes were upregulated after infection of the elicited VA5 and E00 lines, respectively (Supplementary Figure 1C). Some studies show that the induction of an enhanced stress response by stimulation with pathogen virulent strains or elicitors such as SA or BTH is only evident after a subsequent infection with the active pathogen (Mur et al., 1996; Cameron et al., 1999; van Wees et al., 1999; Kohler et al., 2002). In our work, however, none of the genes studied was upregulated when the MeJA-primed lines were challenged with the oomycete as compared to infected controls (Supplementary Figures 1B, C). These results could be explained by a decline in the expression at the time that samples were collected (14 days after elicitation) as described in Milan et al. (2017) or by the suppression of gene expression induced by some metabolites which are produced by the phenylpropanoid pathway, as described in Rubio-Rodríguez et al. (2021). Uncorrelation between terpene metabolite production and the expression of their biosynthesis genes was reported in a MeJA-treated genotype of Norway spruce (Mageroy et al., 2020).

The degree of plant resistance to stress is influenced by systemic signals mediated by phytohormones; thus, SA-mediated signaling triggers resistance against biotrophic and hemi-biotrophic pathogens, while the signal mediated by the combination of JA and ET activates resistance to necrotrophic pathogens. Although JA and SA signaling pathways are currently described as antagonistic, other studies show that JA-mediated defenses are also effective against biotrophic or hemi-biotrophic pathogens (Han and Kahmann, 2019). In our study, ABA, JA, and SA contents were determined in embryogenic lines E00, Q8, and VA5. Elicitation caused a generalized increase in ABA concentration, except in the Q8 line, which basal levels were much higher than those from the other two lines; infection induced an ABA decrease, only significant in the samples that had been previously elicited. ABA is involved in the initiation of adaptative responses to various environmental conditions, although in relation to disease resistance its effect has been considered to be negative (Mauch-Mani and Mauch, 2005) which may be explained by the role of ABA in the inhibition of phytoalexin biosynthesis and *PAL* transcripts accumulation, in addition to being used by pathogens as an effector molecule (Lievens et al., 2017). There is also evidence that ABA interferes at different levels with the biotic stress response pathways mediated by SA and JA/ET (de Torres Zabala et al., 2009). However, several studies report a positive effect of ABA accumulation in terms of resistance to diseases caused by viruses, due to its ability to inhibit the transcription of a β-1,3-glucanase, forming a physical barrier against the spread of the virus (Whenham et al., 1986; Beffa et al., 1996; Rezzonico et al., 1998), but in their interaction with pathogen fungi and oomycetes ABA accumulation compromises host resistance (Mauch-Mani and Mauch, 2005).

Elicitation with MeJA increased the JA content of holm oak Els, and infection induced a dramatic increase in the content of this hormone only in these samples that had been previously elicited (Figure 1B), while levels of JA of not elicited samples were similar to those of the uninfected control. It has been reported that the JA-mediated defense pathway is activated by infection with necrotrophic pathogens (Thomma et al., 1998; Glazebrook, 2005), which promote the biosynthesis of this hormone, triggering the stress response (Ramirez-Estrada et al., 2016). Corroborating this, Long et al. (2021) reported the requirements of jasmonate signaling for defense responses against *Phytophthora nicotianae* in tobacco, which included the phenylpropanoid and the sesquiterpenoid biosynthesis pathways. In Norway, spruce accumulation of JA and not SA following wounding of MeJA-treated bark has also been reported (Mageroy et al., 2020) and authors suggest that MeJA primes JA-dependent defenses in the species although they found difficulties to link the response with expression pattern of PR proteins or JA biosynthesis enzymes. We also suggest that the observed increase in JA content could be considered as a marker of the activation of the defense response to the pathogen, since it is common in the three lines tested.

Holm oak ELs contents in SA varied among the different lines, and increased by elicitation but were not altered after infection (Figure 1C). This could indicate that elicitation might activate several defense pathways, while the response to the infection with this necrotrophic pathogen is mediated by the JA signaling pathway. However, a synergistic behavior between both hormones has been reported in the response to abiotic and biotic stresses (Yang et al., 2019). Also, some effectors from necrotrophic and biotrophic fungal pathogens have probed to alter the available pool of chorismate, the precursor of the SA, by different mechanisms such as the isochorismatases produced by *Phytophthora sojae* (Han and Kahmann, 2019). However, our results indicate that SA content was significantly associated to higher expression rates of the *CS* gene, therefore we did not observe this infection mechanism in the pathosystem *Quercus ilex-Phytophthora cinnamomi*.

Plants release a great diversity of phenolic compounds related to stress defense (Rashad et al., 2020), many of them are produced in the phenylpropanoid biosynthesis pathway. These secondary metabolites include flavonoids, monolignols, phenolic acids, stilbenes and coumarins, all of them playing an essential role in the vital development of plants as essential components of cell walls, and as phytoalexins against herbivores and pathogens, as well as protectors against intense light and UV radiation (Sharma and Gautam, 2019; Wallis and Galarneau, 2020). Our study analyzed the variation in the phenolic profile of E00, Q8, and VA5 lines, determining the content of phenolic acids (caffeic, ellagic, ferulic, and sinapic acids), the flavanol quercetin and the proanthocyanin prodelphinidin B3. The phenolic profile of the VA5 line, generated from a tolerant tree, differed from the other two lines. It is worth noting the high caffeic acid content detected in this line, which can be considered as a marker of defense that was also detected in susceptible lines after eliciting with MeJA (Figure 2A), along with increased levels of sinapic and ferulic acids and of quercetin in the E00 line. Caffeic acid and its derivatives are considered secondary metabolites of great importance for actively participating in the defense mechanism of plants against biotic stress (Riaz et al., 2018). This acid is involved in the synthesis of lignin, which allows a strengthening of the cell wall, and it also inhibits the synthesis of proteins in pathogenic cells, while increases in caffeic acid activate the production of enzymes that have a role in the inhibition of pathogens (Osbourn, 1996; Davidson, 1997; Riaz et al., 2018). We found that expression of the *PAL* gene was inversely correlated to caffeic acid content (μ = −0.351, *p* = 0.036) probably due to a transcriptional inhibition of the *PAL* gene exerted by the metabolite (Zhang and Liu, 2015). Overall, elicitation with MeJA caused significant increases in sinapic, ferulic, and caffeic acids and in quercetin (Figure 3). This increased production of phenol derivates was also obtained when applying elicitors to roots of bitter melon (Chung et al., 2016) and stevia plants (González-Chavira et al., 2018). Finally, sterile filtrates of *Verticillum non-alfalfae* significantly increased *PAL* activity in hop cell lines (Kunej et al., 2020). In our assays, infection decreased caffeic acid content in control ELs, suggesting that the oomycete infection inhibits the expression of some genes related to tannin biosynthesis, as was described in holm oak (Gallardo et al., 2019). These authors reported that mechanical defoliation (considered an abiotic stress) increased the transcription of genes related to the synthesis of tannins, while infection with the oomycete would inhibit its expression, being partly responsible for the dieback symptoms. Therefore, the increased contents in these phenolic acids observed in elicited holm oak ELs could suppose a defense mechanism to subsequent infections. In other studies, infection with *Phytophthora parasitica* or *Xanthomonas campestris* of plant tissues treated with lipopolysaccharides from these pathogens resulted in significant increases in the production of phytoalexins, flavonoids, and phenolic conjugates (Newman et al., 2002). Therefore, the increase in ellagic acid after infection in elicited samples can be considered as a marker of stress response, being common in all three lines.

There are few reports on the effect of priming on forest tree species. Indeed, most of them are related just to one genotype (as an example see Mageroy et al., 2020). Here we report on work done with 3 holm oak genotypes (including one derived from a “scape” tree, VA5) and found phenotypic differences, therefore demonstrating that genotypes might display different strategies (gene expression, metabolite and hormonal profiles) after pathogen infection, which supports the complexity of the plant-pathogen interaction. Our work identifies changes triggered by MeJA elicitation in holm oak embryogenic lines, characterized by increases in ABA and JA contents, as well as in levels of most of the determined phenolic compounds, especially in caffeic acid, but not in their biosynthesis genes. The response to oomycete infection in holm oak elicited plant material is characterized by a further increase in JA. Since JA and phenols have been described as a part of the *Q. ilex* defense response against *P. cinnamomi*, we propose that MeJA may act as an IR stimulus. In our previous work (Morcillo et al., 2020), we performed dual cultures with MeJA- elicited ELs confronted to *P. cinnamomi* mycelium, and we referred increases in H_2_O_2_ content, therefore indicating an activation of defense responses. In conclusion, MeJA induced in our embryogenic material both direct (detected prior to any challenge) and primed (detected after subsequent challenge) defense responses. Further studies focused on the underlying mechanisms involved in this primed defense response need to be addressed.

## Data Availability Statement

The original contributions presented in the study are included in the article/Supplementary Material, further inquiries can be directed to the corresponding author/s.

## Author Contributions

IA, ES, and JS contributed to conception and design of the study. ES and MM performed the statistical analysis. MM, EC, MTM, and ES performed the experiments. MM, ES, and IA wrote the first draft of the manuscript. MM, MTM, EC, JS, IA, and ES wrote sections of the manuscript. All authors contributed to manuscript revision, read, and approved the submitted version.

## Funding

This work was supported by the research project co-financed by MICINN and the EU (AGL2013-47400-C4-04-R, AGL2016-76143-C4-01-R, AGL2016-76143-C4-04-R, and PID2020112627RB) and by a predoctoral contract to MM (BES-2014-069171).

## Conflict of Interest

The authors declare that the research was conducted in the absence of any commercial or financial relationships that could be construed as a potential conflict of interest.

## Publisher's Note

All claims expressed in this article are solely those of the authors and do not necessarily represent those of their affiliated organizations, or those of the publisher, the editors and the reviewers. Any product that may be evaluated in this article, or claim that may be made by its manufacturer, is not guaranteed or endorsed by the publisher.

## Supplementary Material

Supplementary Figure 1 Relative gene expression (2^−ΔΔCt^) of *Chorismate Synthase (CS), Phenylalanine-Ammonia-Lyase (PAL), NADPH-dependent Cinnamyl Alcohol Dehydrogenase (CAD)*, and *Chalcone Synthase (CHS)* genes in embryogenic lines from three holm oak genotypes (E00, Q8, and VA5) after elicitation with MeJA **(A)**, and after infection with *Phytophthora cinnamomi* of control **(B)**, or elicited plant material **(C)**. For each genotype and gene, data are mean ± SE of ratios of gene expression estimated from triplicated amplifications performed with three biological replicates. Values above or below 1 denote up- or downregulation of the gene, respectively; ^*^Denotes significant effect of the treatment as compared to the reference sample, either control **(A,B)** or elicited **(C)** plant material.
