# Supplementary figures and images for: Effect of Methyl Jasmonate in Gene Expression, and in Hormonal and Phenolic Profiles of Holm Oak Embryogenic Lines Before and After Infection With Phytophthora cinnamomi
Source: Front Plant Sci. 2022 Mar 9;13:824781. doi: 10.3389/fpls.2022.824781 (PMC8959775; doi:10.3389/fpls.2022.824781)

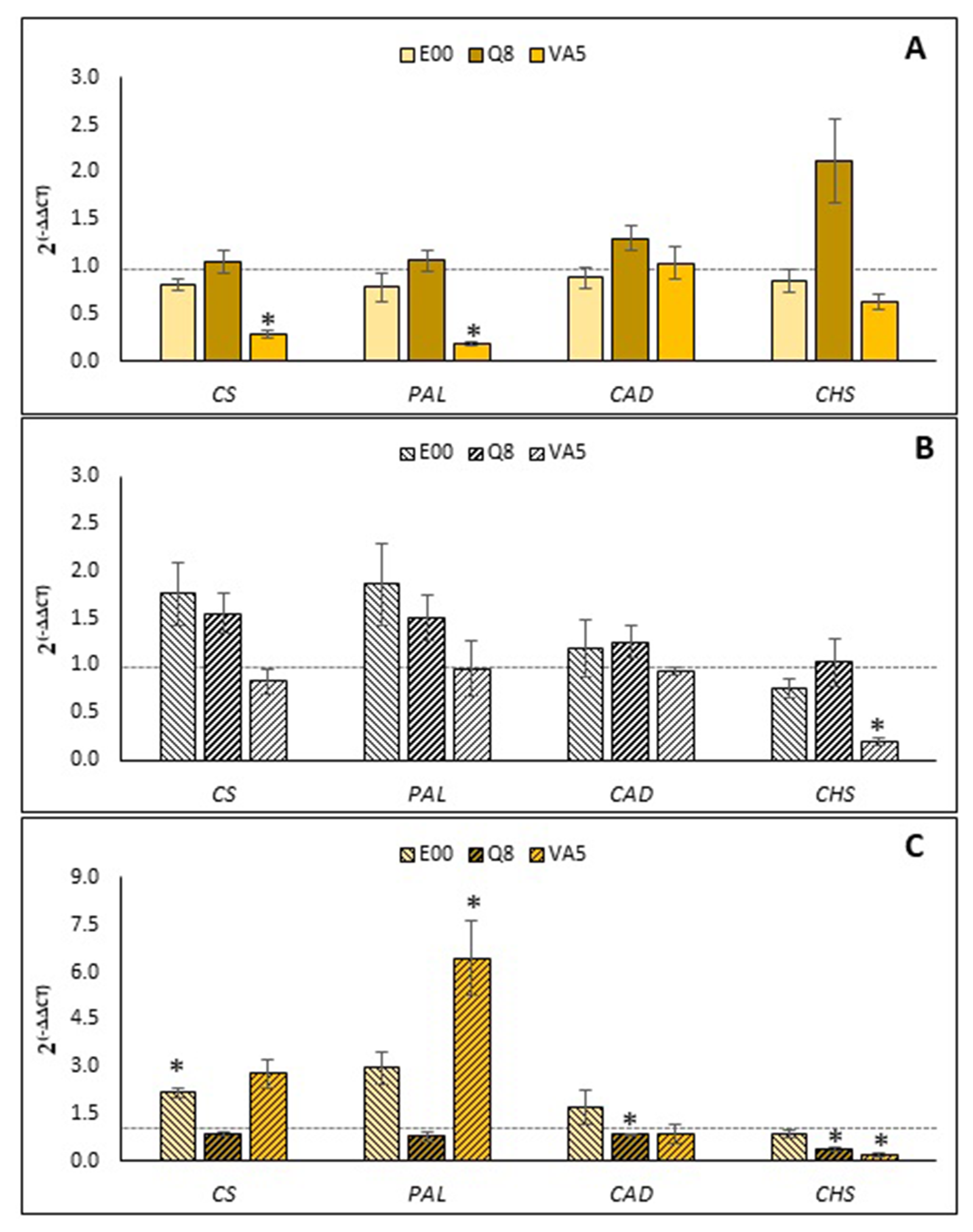

Supplement: Supplementary Figure 1 — Relative gene expression (2−ΔΔCt) of Chorismate Synthase (CS), Phenylalanine-Ammonia-Lyase (PAL), NADPH-dependent Cinnamyl Alcohol Dehydrogenase (CAD), and Chalcone Synthase (CHS) genes in embryogenic lines from three holm oak genotypes (E00, Q8, and VA5) after elicitation with MeJA (A), and after infection with Phytophthora cinnamomi of control (B), or elicited plant material (C). For each genotype and gene, data are mean ± SE of ratios of gene expression estimated from triplicated amplifications performed with three biological replicates. Values above or below 1 denote up- or downregulation of the gene, respectively; *Denotes significant effect of the treatment as compared to the reference sample, either control (A,B) or elicited (C) plant material. [file Image_1.tif]
